# Smartphone multiplex microcapillary diagnostics using Cygnus: Development and evaluation of rapid serotype-specific NS1 detection with dengue patient samples

**DOI:** 10.1371/journal.pntd.0010266

**Published:** 2022-04-07

**Authors:** Sarah Helen Needs, Sirintra Sirivisoot, Sophie Jegouic, Tanapan Prommool, Prasit Luangaram, Chatchawan Srisawat, Kanokwan Sriraksa, Wannee Limpitikul, Dumrong Mairiang, Prida Malasit, Panisadee Avirutnan, Chunya Puttikhunt, Alexander Daniel Edwards

**Affiliations:** 1 Reading School of Pharmacy, University of Reading, Whiteknights, Reading, United Kingdom; 2 Division of Dengue Hemorrhagic Fever Research, Faculty of Medicine Siriraj Hospital, Mahidol University, Bangkok, Thailand; 3 Molecular Biology of Dengue and Flaviviruses Research Team, Medical Molecular Biotechnology Research Group, National Center for Genetic Engineering and Biotechnology, National Science and Technology Development Agency, Pathum Thani, Thailand; 4 Department of Biochemistry, Faculty of Medicine Siriraj Hospital, Mahidol University, Bangkok, Thailand; 5 Pediatric Department, Khon Kaen Hospital, Ministry of Health, Khon Kaen, Thailand; 6 Pediatric Department, Songkhla Hospital, Ministry of Health, Songkhla, Thailand; 7 Siriraj Center of Research Excellence in Dengue and Emerging Pathogens, Faculty of Medicine Siriraj Hospital, Mahidol University, Bangkok, Thailand; 8 Capillary Film Technology Ltd, Billingshurst, West Sussex, United Kingdom; US Department of Homeland Security, UNITED STATES

## Abstract

Laboratory diagnosis of dengue virus (DENV) infection including DENV serotyping requires skilled labor and well-equipped settings. DENV NS1 lateral flow rapid test (LFT) provides simplicity but lacks ability to identify serotype. A simple, economical, point-of-care device for serotyping is still needed. We present a gravity driven, smartphone compatible, microfluidic device using microcapillary film (MCF) to perform multiplex serotype-specific immunoassay detection of dengue virus NS1. A novel device–termed Cygnus–with a stackable design allows analysis of 1 to 12 samples in parallel in 40 minutes. A sandwich enzyme immunoassay was developed to specifically detect NS1 of all four DENV serotypes in one 60-μl plasma sample. This test aims to bridge the gap between rapid LFT and laboratory microplate ELISAs in terms of sensitivity, usability, accessibility and speed. The Cygnus NS1 assay was evaluated with retrospective undiluted plasma samples from 205 DENV infected patients alongside 50 febrile illness negative controls. Against the gold standard RT-PCR, clinical sensitivity for Cygnus was 82% in overall (with 78, 78, 80 and 76% for DENV1-4, respectively), comparable to an in-house serotyping NS1 microplate ELISA (82% vs 83%) but superior to commercial NS1-LFT (82% vs 74%). Specificity of the Cygnus device was 86%, lower than that of NS1-microplate ELISA and NS1-LFT (100% and 98%, respectively). For Cygnus positive samples, identification of DENV serotypes DENV2-4 matched those by RT-PCR by 100%, but for DENV1 capillaries false positives were seen, suggesting an improved DENV1 capture antibody is needed to increase specificity. Overall performance of Cygnus showed substantial agreement to NS1-microplate ELISA (κ = 0.68, 95%CI 0.58–0.77) and NS1-LFT (κ = 0.71, 95%CI 0.63–0.80). Although further refinement for DENV-1 NS1 detection is needed, the advantages of multiplexing and rapid processing time, this Cygnus device could deliver point-of-care NS1 antigen testing including serotyping for timely DENV diagnosis for epidemic surveillance and outbreak prediction.

## Author summary

Diagnosis of the important mosquito-transmitted dengue virus (DENV) requires laboratory assays to detect viral genome (RT-PCR), viral NS1 protein (immunoassay) or DENV specific antibodies. Current point-of-care NS1 tests cannot distinguish serotype, so laboratory tests are still essential to determine which of 4 DENV serotypes is present. Here we present a rapid serotype-specific NS1 test in a portable microfluidic format. Ten parallel 0.2 mm tubes inside a flat plastic ribbon perform multiplex NS1 immunoassays. A simple cassette delivers sample and reagents sequentially through the microcapillaries by gravity. By stacking cassettes, 12 tests could be performed in under 40 minutes, with results recorded by smartphone. When evaluated with 205 patients plus 50 control samples, and results compared to conventional RT-PCR, the sensitivity for DENV1 to 4 was 78%, 78%, 80%, and 76%, respectively, with specificity of 100% for DENV2-4. DENV1 showed some false positives due to cross-reactivity of the capture antibody. Serotyping performance with MCF-Cygnus devices showed substantial agreement to the serotyping-NS1 microplate ELISA. Therefore, these simple and portable microcapillary immunoassay devices could support dengue NS1 serotyping with potential benefits for near-patient diagnosis, real-time epidemic surveillance and outbreak mapping.

## Introduction

Dengue virus (DENV) is a member of *Flavivirus* genus, which shares some typical RNA genomes with West Nile virus, Zika virus, yellow fever virus, and Japanese encephalitis virus. There are nearly estimated 400 million cases of dengue annually, being endemic in tropical and subtropical countries and efficiently transmitted by mosquitoes, mostly *Aedes* spp. [1]. DENV genomes contain of three structural (capsid (C), pre-membrane protein (prM), and envelope (E)) and seven non-structural proteins (NS1, NS2A, NS2B, NS3, NS4A, NS4b, and NS5) [2]. During viral replication in an infected host, soluble NS1 proteins are secreted in high concentrations into the circulation and to a lesser extent saliva and urine [3,4,5]. NS1 from four serotypes of DENV is used as a common diagnostic marker (up to nine days after onset) in both acute primary and secondary infections [6]. Many commercial DENV NS1 tests are available, based on rapid immunochromatography (lateral flow rapid tests used at point-of-care) and microplate ELISA (in diagnostic laboratory), such as Panbio, Standard Diagnostics, and Bio-Rad. Their overall sensitivity and specificity varied from 45 to 80% and 93 to 100%, respectively [7,8,9,10]. Both methods use only small volumes of sample (blood/serum/plasma) (50–100 μL). When comparing the processing times, the one-step sandwich-format microplate ELISA could provide a result in 120 min, while rapid tests need less than 30 min. Even though the rapid test is faster, lateral flow immunoassays have a much higher limit of detection compared to a microplate ELISA and can therefore only detect higher levels of viral protein. At antigen levels approaching the limit of detection, visual interpretation of lateral flow rapid tests can be ambiguous. However, lateral flow test readers are increasingly available to improve result interpretation [11]. Microplate ELISA avoids visual interpretation as this is routinely measured with a microplate reader but requires laboratory operation. Therefore, a need for higher sensitivity rapid tests that can be performed with minimal laboratory equipment is required.

The antibody pairs in DENV NS1 immunoassays that are currently in clinical use detect NS1 from all four serotypes with varying analytical sensitivity. However, by careful selection of antibody clones that bind to conserved epitopes, serotype-selective immunoassays have been developed including microplate ELISA [12,13,14]. A prototype lateral flow immunoassay could differentiate each DENV serotype, and also distinguish DENV from Zika virus without cross reactivity [15], offering the potential for dengue serotyping rapid tests, although NS1 from Zika virus is found at much lower plasma concentrations making lateral flow unlikely to be sensitive enough for Zika diagnosis. This rapid test panel showed sensitivity and specificity to each DENV NS1 serotype ranging from 76–100%, illustrating the potential for serotype-specific antibody pairs to define serotype from plasma NS1.

When patients have a secondary infection caused by a different DENV serotype they may be more prone to develop more severe forms of disease—dengue hemorrhagic fever (DHF) and dengue shock syndrome (DSS)—due to antibody-dependent enhancement (ADE) [16]. If severe dengue patients do not receive appropriate treatment or immediate hospitalization, fatalities are possible. The effect of serotype infection on patient outcome is not fully understood since serotyping is mainly used for surveillance. However, the serotype of secondary infections may indicate disease outcomes [17]. If this could be accurately mapped it is possible that serotype-specific rapid tests could be prognostic for severe disease, however, knowledge of concurrent or secondary infection currently provides better prediction of severe disease [18]. Rapid knowledge of infecting DENV serotype has potential value for early intervention, for disease surveillance and for epidemic control. To identify four serotypes of DENV, conventional virus isolation and characterization, and RT-PCR methods are currently performed [19,20,21]. Within the past decade, microplate DENV serotyping-NS1 ELISAs have also been developed as in-house assays, but prediction of some serotypes was still inaccurate [12,13,14]. Our recent modified serotyping-NS1 microplate ELISA (plus) showed perfect concordance in four serotypes identification in dengue patients’ specimens comparing to RT-PCR [22], indicating that immunoassay methods are capable of accurate serotyping. An ideal DENV diagnostic tool would need to be as sensitive and specific as possible, be able to be interpreted in real-time, should be easy to perform without investment in laboratory instruments, cheap to manufacture, and capable of classifying DENV serotypes. Real-time monitoring of dengue serotype infection will also be helpful for epidemiology, disease surveillance, and public health strategy to control endemic infection and target interventions (e.g., mosquito control) to epidemic outbreaks [23].

Microfluidic technology can fabricate miniature compact bioassay devices offering many benefits to healthcare systems across the world if economical, robust, and reproducible fabrication can be achieved. Microfluidic and lab-on-a-chip devices have been developed to screen, detect, or monitor tumor cells and to measure and detect various protein and nucleic acid biomarkers in human biological samples [24,25,26]. Microcapillary film (MCF) has been shown to be useful for miniaturizing sandwich ELISA to detect biomarkers in clinical samples [27,28,29,30,31]. Produced by melt-extrusion of fluorinated ethylene propylene (FEP), MCF comprises a flat ribbon containing ten parallel microcapillaries. This fluoropolymer plastic film has optical clarity from refractive index matching allowing the bioassay output (color or fluorescence) to be easily observed and simply quantified on a smartphone camera [29,32]. In previous studies, MCF devices have been developed, using an ELISA-based assay format, in both single and multiplex assay strips that were driven by pressure (using a syringe to deliver sample and reagent) and interpreted by a flatbed scanner [27,28] or a smartphone camera [29]. For simpler assays requiring only a sample plus single reagent, MCF test strips used capillary action achieved by coating the inner surface with hydrophilic polymer [33]; this hydrophilic polymer coating is compatible with antibody coating for immunoassays [31,32,33]. Recently, we further reduced the complexity of MCF immunoassay devices by using gravity to flow the sample followed by wash and detection reagents sequentially through microcapillaries [34].

Previous work on protein biomarker detection has identified that immunoassays using MCF devices performed as well as microplate ELISA [30,31], but only with spiked simulated samples. Here, we developed multiplex MCF immunoassays for viral antigen and for the first time evaluated the analytical performance of the tests with human clinical patient samples. We described the transfer of sandwich immunoassays from microplate ELISA to MCF test strips to develop a simple rapid assay method for the detection of serotype specific DENV NS1. We used gravity driven MCF devices to detect and differentiate multiplex serotype-specific DENV NS1 proteins in 205 undiluted patient plasma samples alongside 50 other febrile illness controls. MCF assays were performed using a novel holder, termed Cygnus, that simplified sample and reagent addition to MCF. The Cygnus device can be stacked in microplate compatible spacing to test up to 12 samples (i.e., 120 individual immunoassays) and showed stacked Cygnus devices permit easy-to-use and flexible rapid multiplexed immunoassays in MCF.

## Methods

### Ethics statement

This study was approved by the ethics committee of Siriraj Institutional Review Board, Faculty of Medicine Siriraj Hospital, Mahidol University, Thailand (EC 593/2561). The written formal consent was obtained from parents or guardians before enrollment of each patient.

### Clinical samples

Retrospective clinical specimens collected from pediatric patients (aged 2–14 years) of two dengue clinical cohorts in Khon Kaen and Songkhla hospitals, Thailand, during year 2002 to 2012, were used in this study. The specimens from individual enrolled patients were consecutively collected at different time points every day from the date of admission during the onset of fever until defervescence and discharge, together with the 2-week, 2-month and 6-month follow ups. To determine dengue immune status, paired specimens of each patient (including acute-febrile phase at admission date and 2-week convalescent samples) were analyzed for anti-DENV IgM and IgG antibodies by IgM/IgG capture microplate ELISA [35] with some modification. Primary infection was interpreted in patients who gave IgM rising titer (seroconversion) and a ratio of IgM/IgG titer over 1.8 in convalescent samples. In contrast, secondary infection was identified in patients who achieved IgG rising titer and a ratio of IgM/IgG titer less than 1.8. DENV infecting serotypes were determined in admission acute specimens by multiplex RT-PCR [36]. Patients who were negative by RT-PCR and no seroconversion in pair specimens were identified as other febrile diseases or non-dengue cases (S1A Fig). According to WHO 1997 guideline, clinical severity of dengue infection was classified as mild dengue fever (DF) to severe dengue hemorrhagic fever (DHF) [37]. DF was characterized by clinical symptoms of fever with at least two other clinical findings such as hemorrhagic manifestation and thrombocytopenia. DHF included evidence of plasma leakage leading to shock and death if timely intervention is not available. Sample size used in this study was calculated based on expected sensitivity and specificity at 85% with an acceptable margin of error of ±10%. The confident level was set at 95% (i.e., alpha = 0.05) [38]. Two-hundred and five retrospective DENV-infected acute plasma including four serotypes were randomly selected from dengue clinical bank. They consisted of 50 samples of DENV1, 55 samples of DENV2, 50 samples of DENV3, and 50 samples of DENV4. The majority of the samples were identified as secondary infection (98%; 4 primary and 201 secondary). The numbers of DF/DHF patients were 84/121 (S1B Fig). Another 50 samples from patients with other febrile diseases were also included in this study as non-dengue specimens. The samples were processed retrospectively and were stored at -70°C until use.

### Multiplex RT-PCR for DENV serotyping

DENV serotyping was performed as previously described [36]. Briefly, isolated DENV RNA from patient samples were reverse-transcribed to generate first-strand cDNA. This was amplified by PCR using pan-dengue primers and four pairs serotype-specific primers. DENV serotype was based on the RT-PCR product molecular size when separated by agarose gel electrophoresis. DENV serotypes were compared with DENV 1, 2, 3 and 4 standards (506, 346, 196 and 143 bp, respectively).

### DENV Serotyping-NS1 Microplate ELISA

The serotyping NS1 microplate ELISA was developed as described [22]. Briefly, each plasma sample (1:5 dilution) was added to four wells pre-coated with the pan-serotype capture antibody (2E11). Biotinylated serotype-specific antibodies were added to each well separately. In some cases, an additional assay using the antibody pair 2E11/5F3 (for DENV1 and DENV3 subcomplex) was used to increase sensitivity of the assay [22]. Positive samples were classified when the assay OD reading exceeded twice the value of the negative plasma control. DENV serotype prediction was based on the antibody pair which gave the highest OD reading.

### Dengue NS1 Ag rapid test

SD BIOLINE Dengue Ag Rapid Test (Abbot, FL, USA) was used as a representative rapid lateral flow test (LFT). Samples were processed according to the manufacturer’s instructions. Briefly, 100 μL plasma was added to the sample well and incubated for 15–30 min. Positive cases were assessed by eye and interpreted by the appearance of both test and control lines.

### MCF based cygnus device ELISA

**Fig 1:**
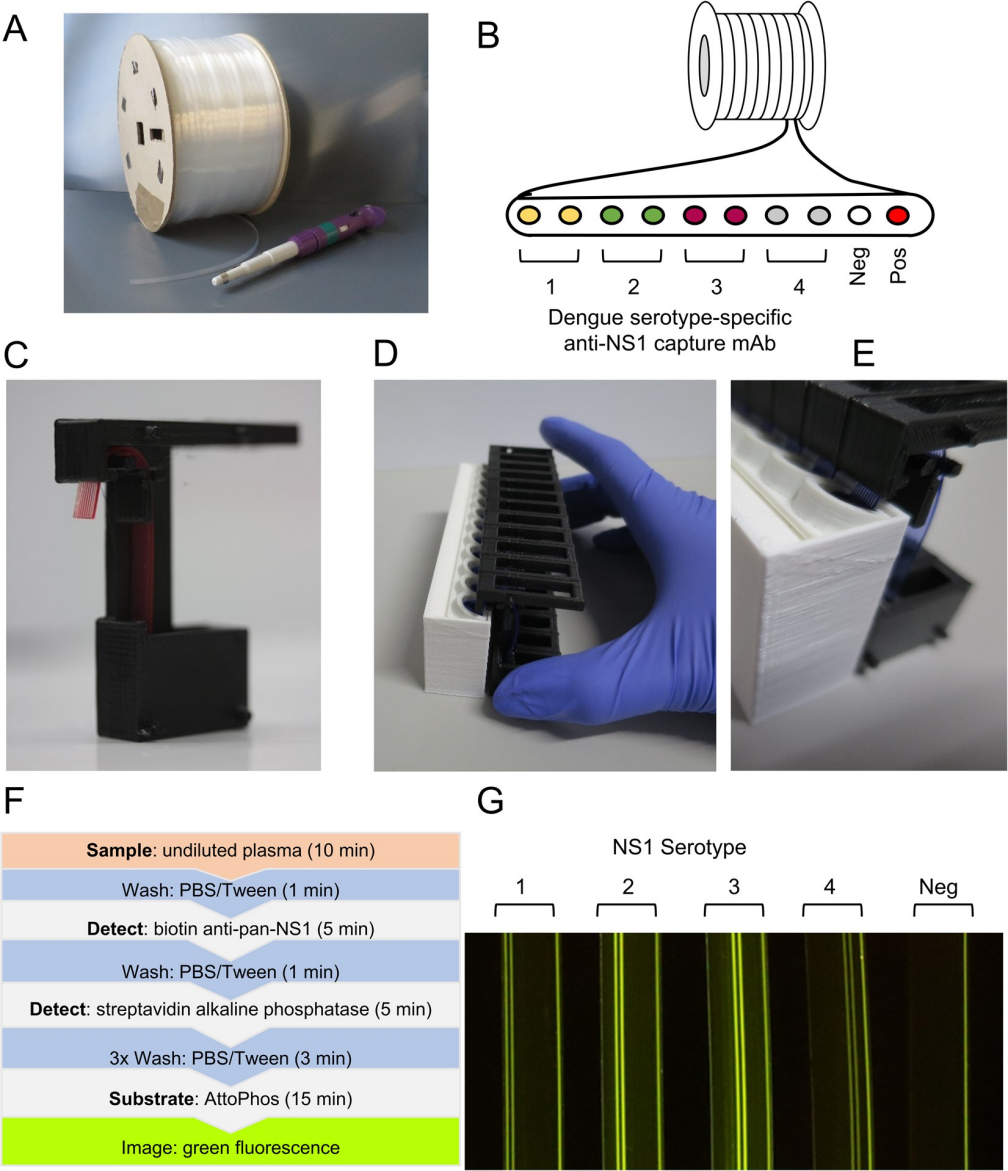
Multiplex serotype-specific NS1 microcapillary immunoassay using stackable Cygnus devices. (A) Reel of 0.5 Km of Microcapillary film. (B) Individual capillaries can be coated in bulk before being cut to 47 mm test strips. Duplicate capillaries were coated with serotype specific antibodies. Negative capillary was left uncoated and the positive capillary were coated with NS1 antigen. (C) Single gravity driven cassette with MCF filled with blood. (D) A block of 12 Cygnus cassettes clipped together with the end of the MCF located in the strip well. (E) A block of Cygnus cassettes interfaced with the custom stripwell. (F) Flow diagram of assay steps. (G) Individual antigens (25 ng/mL) were spiked into undiluted heparinised-plasma.

Duplicate capillaries of the MCF material were coated with serotype-specific capture antibodies (Fig 1A and 1B) described in Table 1. Sample processing steps and a detailed method is described in the supporting information (S1 Text). Briefly, all steps used 60 μL volume of sample, reagent, and wash buffer. Undiluted patient plasma was added to the wells. The MCF test strips in the Cygnus device were dipped into the wells (Fig 1C–1E) and incubated for 10 mins. Serotype for patient plasma was unknown to researchers conducting the tests. Subsequent washes, biotinylated pan-serotype detection antibody, streptavidin-alkaline phosphatase and AttoPhos were added to fresh wells and the MCF dipped into each reagent subsequently (Fig 1F). The siphon design moved the reagents through the capillary film by gravity. Patient samples were processed in batches of 6–12 samples at a time (Fig 1D).

**Table 1:** DENV NS1 antibodies used in the Cygnus test.

Antibody	Reactivity to DENV serotype	Type of epitope	Isotype
84B	DENV1	Conformation	IgG1
1B10	DENV2	Linear	IgG1
46A	DENV3	Conformation	IgG2a
4A	DENV4	Conformation	IgG2b
1F11	Pan-serotype DENV1-4	Linear	IgG2a

### Surface Plasmon Resonance (SPR)

Affinity and Kinetic binding between NS1 proteins and anti-NS1 antibodies were analyzed by Surface Plasmon Resonance technique using Biacore X100 (GE Healthcare, USA) as described [22]. The sensorgram and affinity constant (K_D_) were calculated by Biacore X100 software. Three replications were performed from each DENV serotype.

### Image and data analysis

Fluorescence of converted substrate was captured by smartphone or digital camera, found previously to be equally suitable to quantify assay signal [39]. The images were split into red, green, and blue (RGB) channels by ImageJ software (NIH, MD, USA). Only the green channel intensity was analyzed, where fluorescence signal was maximal, with the maximum intensity of each capillary recorded and then normalized to the average intensity of the 5 mM reference strip and reported as relative fluorescence intensity (RFI). An RFI value of 1 therefore represented the enzyme fluorescence signal intensity for a test sample being equal to 5 mM fluorescein. The limit of detection (LOD) was calculated using 3.3 x Std. error of Y-intercept/Slope, while limit of quantitation (LOQ) used a value of 10 x Std. error of Y-intercept/Slope. LOD and LOQ for detection of NS1 target in these assays were calculated from five replica calibration curves of recombinant NS1 spiked at a range of known concentrations into neat negative plasma, with assay performance values calculated using Prism version 8.0 (Graph Pad software, CA, USA).

**Fig 2:**
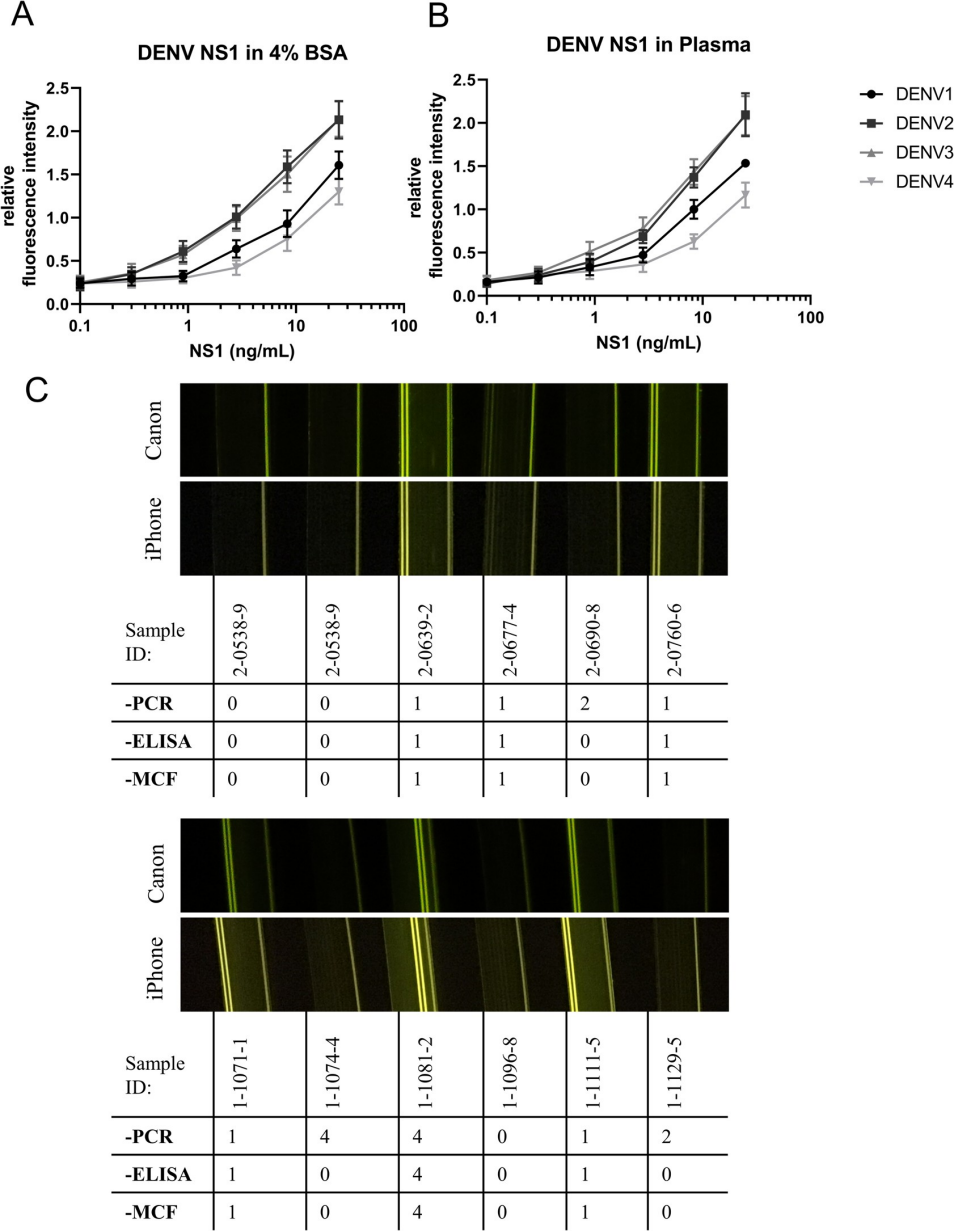
Standard curves for Dengue NS1 detection by Cygnus devices, and examples of dengue patient samples. Serial dilutions of recombinant NS1 of the indicated serotypes was spiked into (A) 4% BSA or (B) plasma and tested with multiplex MCF in Cygnus devices, *n = 4*, error bars indicate ± SEM. (C) A set of patient plasma samples were tested in batches of 6 or 12, and images of two example batches of 6 patient samples are shown. The dengue serotype determined by RT-PCR, conventional microplate ELISA and Cygnus are indicated underneath each sample.

Duplicate capillaries of coated MCF dipsticks that showed mean RFI more than the cutoff value were noted as DENV positive to that serotype. In the case of more than one serotype intensity greater than the threshold, the serotype with most intense signal was reported. The cutoff values from each DENV serotype were calculated by receiver operating characteristic (ROC) analysis and selected using the highest Youden’s index, which noted sensitivity and specificity (Fig 2). Evaluation of the developed test performance was assessed with a standard RT-PCR method. Sample categorization of DENV serotypes for RT-PCR, Serotyping NS1 microplate ELISA, Cygnus device and rapid LFT is detailed in the supporting information (S1 Data). Sensitivity and specificity were calculated as described here [40] using Prism. Confidence Intervals were calculated as described [41] using Prism. Inter-rater concordance or agreement between 2 assays was assessed by Cohen’s kappa (Graph Pad Software, Inc. https://www.graphpad.com/quickcalcs/kappa1/). Performance of NS1 tests were compared pairwise by using Mc Nemar’s exact tests (Graphpad Software, Inc. https://www.graphpad.com/quickcalcs/McNemar1.cfm).

## Results

### Development of a stackable device for rapid DENV NS1 microcapillary immunoassays

Here, we developed a new stackable cassette system–termed Cygnus, named because the capillary test strip resembles a swan’s neck ‘sipping’ the reagents–for holding MCF test strips that simplifies operation by driving sequential sample, wash and detection reagents to flow through the capillaries purely by gravity. In contrast, previous studies used syringes to aspirate samples/wash/reagents. A full stepwise illustration of operation is provided in S2 Fig, and fluid mechanics described in [34].

Serotype-specific antibody combinations and assay conditions were optimized for MCF immunoassays using Cygnus devices. To allow serotype-specific multiplexing, the capture antibody must be serotype-specific and then target detected with a pan-serotype detection antibody. The chosen serotype-specific mAbs (Table 1) were highly specific to DENV NS1 with no cross-reactivity to other flaviviruses and a strong affinity to NS1 molecules (S1 Table). Our serotyping-NS1 microplate ELISA using these antibodies has previously been shown to be suitable for serotype-specific NS1 detection in microplates [13,22] but with a different configuration, using the pan-serotype antibody clone for capture. We therefore expected some difference in analytical and clinical performance between the Cygnus MCF device and microplate ELISA to arise from the differing sandwich assay antibody configuration.

Following optimization, standard curves were performed for each serotype by using NS1 at different concentrations spiked into buffer and heparinized plasma (Fig 2A and 2B). LOD and LOQ of the assay were analyzed. LOD of NS1 spiked into buffer or negative heparinized plasma samples were all below 3 ng/mL (Table 2). Tests were imaged with a Canon S120 Powershot digital camera (approximately £200), and also imaged in parallel with two smartphones representing different price points, Sony Xperia L1 (approximately £150), and iPhone 6S (approximately £400). Assays of patient samples were likewise all imaged by digital camera plus smartphones, but for consistency in subsequent analysis the images taken with the Canon S120 Powershot digital camera were selected. Each image captured results of up to 12 MCF strips from a batch, recording 120 immunoassay data points in a single digital photograph.

**Table 2:** Detection and quantitation limit of DENV NS1 by MCF in Cygnus devices.

DENV NS1	4%BSA		Heparinized plasma	
	LOD (ng/ml)	LOQ (ng/ml)	LOD (ng/ml)	LOQ (ng/ml)
Serotype 1	1.18	3.58	0.73	2.22
Serotype 2	1.43	4.33	1.04	3.14
Serotype 3	1.18	3.59	0.96	2.90
Serotype 4	2.78	8.42	1.76	5.32

### Cygnus performance for dengue NS1 detection

**Fig 3:**
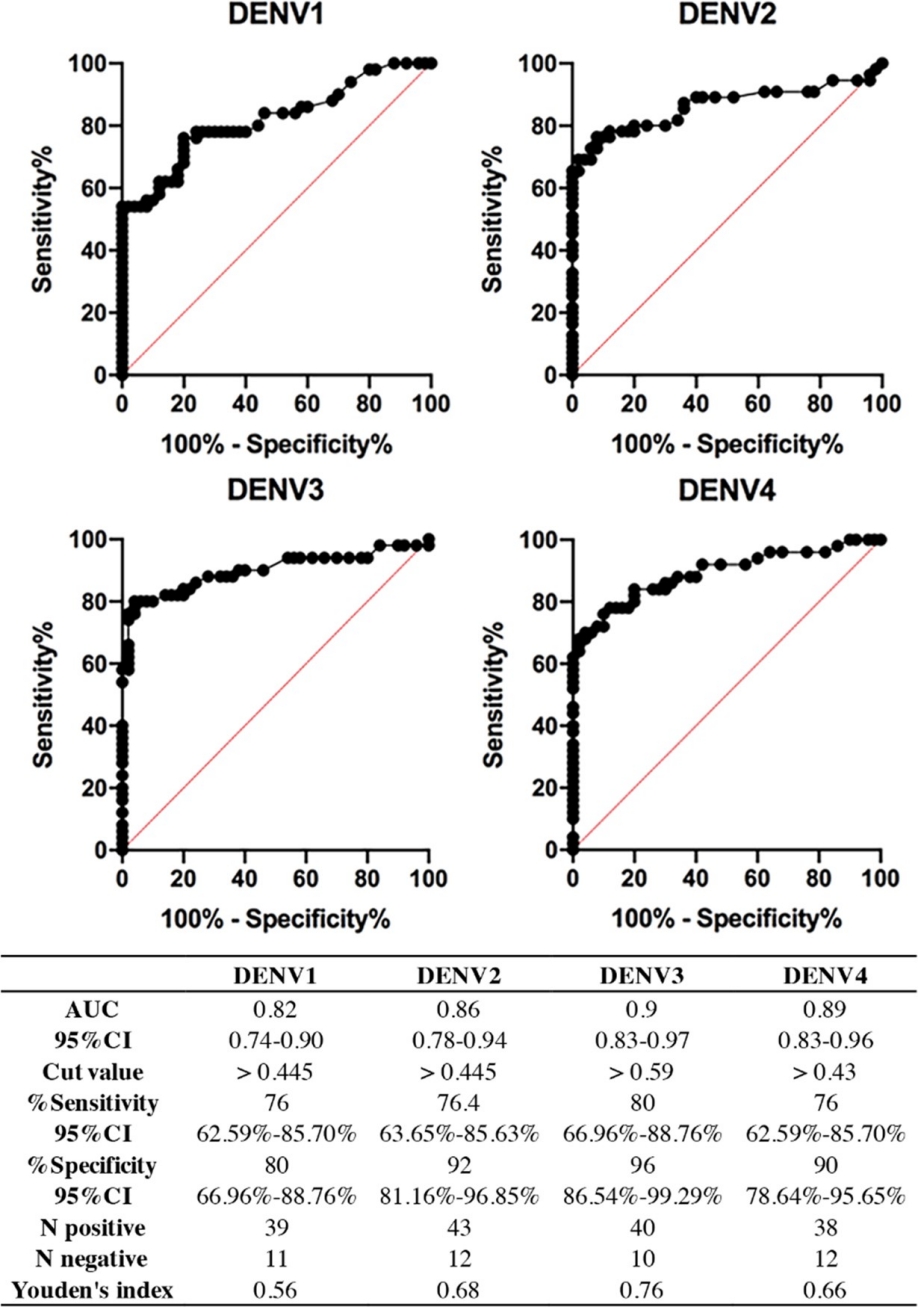
ROC curve analysis for DENV1-4 NS1 detection by MCF in Cygnus devices. A receiver operator characteristic plot for each pair of NS1 capillaries was plotted against PCR determination for the full set of samples vs controls. The table below outlines the key performance indicators for the rapid multiplexed immunoassay devices with this clinical sample set.

All 205 DENV samples plus controls were assessed in the Cygnus device and the images were captured. The mean RFI values of four DENV serotypes obtained by each sample were analyzed. By ROC analysis and Youden’s index, the cutoff values of each serotype were identified in order to retain high sensitivity and specificity (Fig 3). The numbers of positive and negative cases were finally obtained for each serotype.

**Fig 4:**
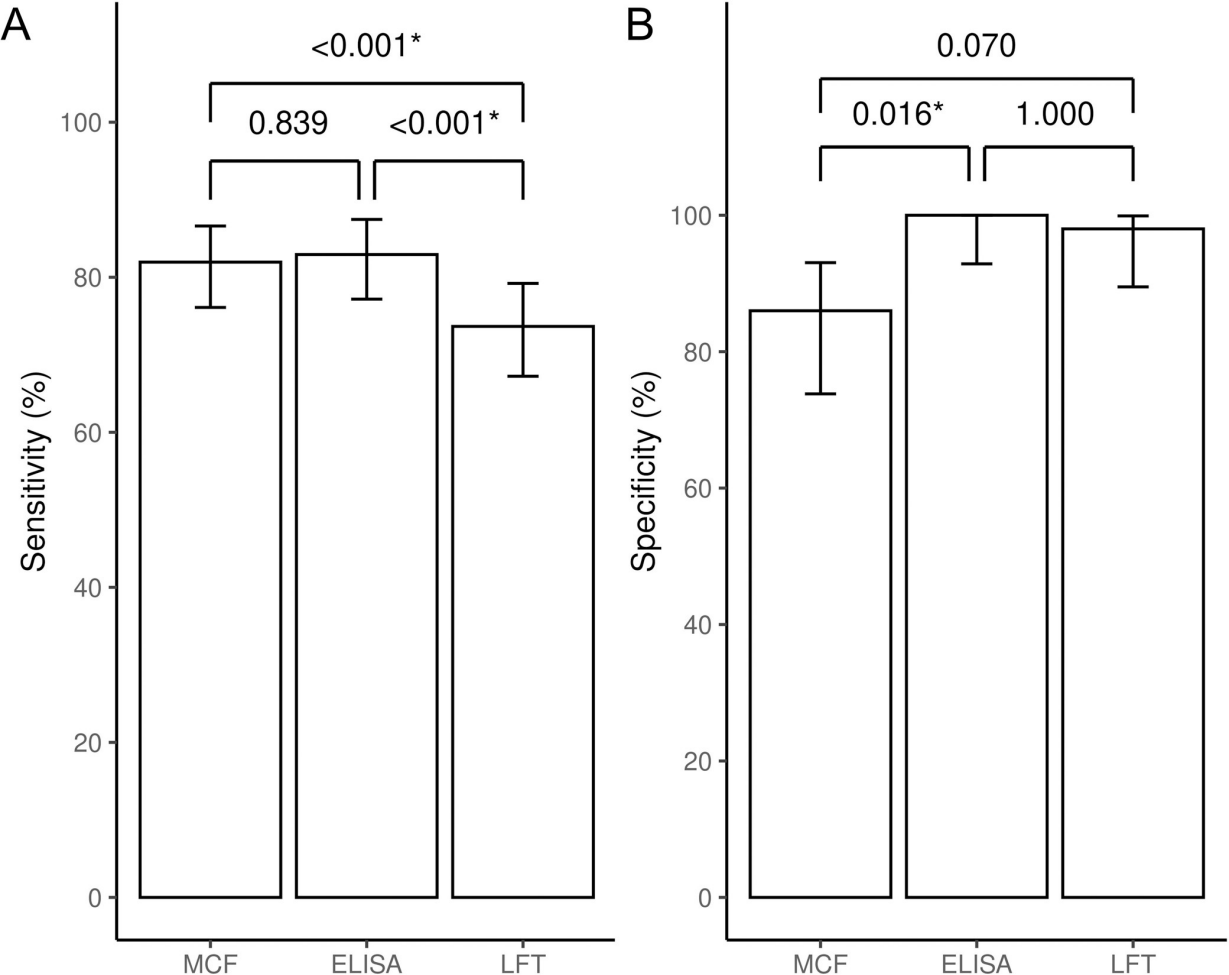
Comparison of detection performances among three NS1 assays. Sensitivities (A) and specificities (B) of MCF (Cygnus), ELISA (5-pairs) and LFT are compared. The error bars show 95 percent confident intervals of the sensitivity and specificity of each assay. The difference in performances is compared pairwise using McNemar’s exact tests. Due to multiple comparisons, the threshold for statistical significance is adjusted to < 0.017 based on Bonferroni correction.

In comparison with standard RT-PCR method, performance of the Cygnus device was evaluated with 255 clinical samples (205 for DENV and 50 for non-DENV). With the selected cutoffs, the Cygnus device achieved 82% sensitivity, 86% specificity and 82.7% accuracy. An agreement between the Cygnus device and RT-PCR was weak (κ = 0.55) (Table 3). In addition, another two different NS1 test formats, DENV serotyping-NS1 microplate ELISA and commercial NS1 lateral flow rapid test (NS1-LFT), were included in this study to compare their performances with the Cygnus device by using the same set of clinical samples (Table 3). Clinical sensitivity of the Cygnus platform was found to be no difference with the NS1 microplate ELISA (82% vs 83%, P = 0.839), but significantly greater than the NS1-LFT sensitivity (82% vs 73.7%, P < 0.001). In contrast, specificity of the Cygnus device (86%) was significantly lower than that of NS1-microplate ELISA (86% vs 100%, P = 0.016), but not significant to NS1-LFT (86% vs 98%, P = 0.070) (Fig 4). It is concluded that the microplate ELISA and Cygnus had equivalent high sensitivity compared to the LFT. In contrast, Cygnus had low specificity compared to both microplate ELISA and LFT. This was due to 7 out of 50 non-DENV samples giving false positives for DENV1 by the Cygnus device. Percentage of accuracy to standard RT-PCR from high to low was 86.3%, 82.7% and 78.4% for microplate ELISA, Cygnus and LFT, respectively, suggesting lower performance of Cygnus than NS1-microplate ELISA, but better than NS1-LFT. The kappa value analysis between NS1-Cygnus and NS1-ELISA (κ = 0.68, 95%CI 0.58–0.77) or NS1-LTF (κ = 0.71, 95%CI 0.63–0.80) indicated that these NS1 assays were in moderate agreement (Table 4). Unlike comparison among NS1 tests, the low level of agreement was found in NS1-Cygnus (κ = 0.55) or NS1-microplate ELISA (κ = 0.66) or NS1-LFT (κ = 0.51) when RT-PCR were compared.

**Table 3:** Overall performances for DENV diagnosis of NS1 Cygnus, NS1-microplate ELISA and NS1-LFT in comparison to standard RT-PCR.

**NS1 assays**		**RT-PCR**			**95%CI**
		**Dengue**	**Non-dengue**	**Total**	
**Cygnus**	**Dengue (n = 175)**	168	7	175	
	**Non-dengue (n = 80)**	37	43	80	
	**Total**	205	50	255	
	%Sensitivity	82% (168/205)			76.11–86.61
	%Specificity	86% (43/50)			73.81–93.05
	% Accuracy	82.7% (211/255)			72.63–93.37
	Agreement (Kappa; κ)*	0.55			0.44–0.67
		**Dengue**	**Non-dengue**	**Total**	
**ELISA**	**Dengue (n = 170)**	170	0	170	
	**Non-dengue (n = 85)**	35	50	85	
	**Total**	205	50	255	
	%Sensitivity	83% (170/205)			77.18–87.46
	%Specificity	100% (50/50)			92.87–100
	% Accuracy	86.3% (220/255)			75.87–97.08
	Agreement (Kappa; κ)*	0.66			0.56–0.76
		**Dengue**	**Non-dengue**	**Total**	
**LFT**	**Dengue (n = 152)**	151	1	152	
	**Non-dengue (n = 103)**	54	49	103	
	**Total**	205	50	255	
	%Sensitivity	73.66% (151/205)			67.23–79.21
	%Specificity	98% (49/50)			89.50–99.90
	% Accuracy	78.4% (200/255)			67.94–89.56
	Agreement (Kappa; κ)*	0.51			0.41–0.61

*Kappa value (level of agreement); 0–0.20 (none); 0.21–0.39 (minimal); 0.40–0.59 (weak); 0.60–0.79 (moderate); 0.80–0.90 (strong); above 0.90 (almost perfect)

**Table 4:** Correlation of NS1-Cygnus performance in comparison with NS1-ELISA or NS1-LFT to detect NS1 in clinical specimens.

NS1-Cygnus	NS1-ELISA			NS1- LFT		
	Dengue	Non-dengue	Total	Dengue	Non-dengue	Total
Dengue	150	17	167	146	28	174
Non-dengue	20	68	88	6	75	81
Total	170	85	255	152	103	255
Agreement (Kappa; κ)*	0.68			0.71		
95%CI	0.58–0.77			0.63–0.80		

*Kappa value (level of agreement); 0–0.20 (none), 0.21–0.39 (minimal); 0.40–0.59 (weak); 0.60–0.79 (moderate); 0.80–0.90 (strong); above 0.90 (almost perfect)

### Cygnus performance for DENV serotyping

In addition to detection of DENV NS1, rapid NS1-Cygnus device was also designed to differentiate DENV serotypes which none of commercial NS1 rapid tests can achieve. Serotype identification of 205 DENV and 50 non-DENV samples by NS1-Cygnus were compared with that by standard RT-PCR (Table 5). Sensitivity of Cygnus to detect NS1 of DENV1-4 was 78%, 78%, 80%, and 76%, respectively. For those that were positive by Cygnus, identification of serotypes was 100% agreement with RT-PCR method in DENV2-4. DENV1 positive samples had 84.78% serotype agreement with RT-PCR (39/46), while another 8 samples (3 of DENV2, 3 of DENV3 and 2 of DENV4) were categorized incorrectly (Table 5). Additionally, the Cygnus test gave 7 false positive results for DENV1 leading to less specificity of MCF-Cygnus (86%) (Tables 3 and 5). These results suggested that DENV1 specific assay requires further optimisation in the Cygnus format. The NS1 microplate ELISA which uses a different antibody configuration showed 100% serotype agreement to the RT-PCR results (Table 6). The NS1-microplate ELISA also showed a higher sensitivity to DENV1 (92% compared to 78%) and DENV3 (88% compared to 80%) than the Cygnus device. However, Cygnus had a higher sensitivity to DENV2 (78.18% compared to 76.36%) and an equal sensitivity to DENV4 (76%) compared to the NS1-ELISA. This might be a result from the additional antibody pair that cross reacted with both DENV1 and DENV3, incorporated to enhance the microplate ELISA assay sensitivity, which was not included in Cygnus device due to the limit in the number of capillaries. This additional antibody pair used in microplates increases the number of assay plates needed from 4 to 5. The results suggested that although the Cygnus device can detect NS1 antigen and identify DENV serotypes in multiplex test strips, it still needs further development to optimise its performance before field evaluation in either hospitals or public health sectors.

**Table 5:** Serotyping performance of NS1-Cygnus compared to RT-PCR in 255 clinical samples.

NS1-Cygnus	RT-PCR				
	DENV1	DENV2	DENV3	DENV4	Negative
DENV1	39	3	3	2	7
DENV2	0	43	0	0	0
DENV3	0	0	40	0	0
DENV4	0	0	0	38	0
Negative	11	9	7	10	43
Total	50	55	50	50	50
**% Sensitivity to serotype**	78% (39/50)	78.18% (43/55)	80% (40/50)	76% (38/50)	
**95% CI**	64.76–87.25	65.63–87.05	66.96–88.76	62.59–85.70	
**% Serotype agreement to PCR**	84.78% (39/46)	100% (43/43)	100% (40/40)	100% (38/38)	
**95% CI**	71.78–92.43	91.80–100	91.24–100	90.82–100	

**Table 6:** Serotyping performance of NS1-microplate ELISA compared to RT-PCR in 255 clinical samples.

NS1-ELISA	RT-PCR				
	DENV1	DENV2	DENV3	DENV4	Negative
DENV1	31	0	0	0	0
DENV2	0	42	0	0	0
DENV3	0	0	38	0	0
DENV4	0	0	0	38	0
Un-identify (DENV1 or 3)	15	0	6	0	0
Negative	4	13	6	12	50
Total	50	55	50	50	50
**% Sensitivity to serotype**	92% (46/50)	76.36% (42/55)	88% (44/50)	76% (38/50)	
**95% CI**	81.16–96.85	63.65–85.63	76.20–94.38	62.59–85.70	
**% Serotype agreement to PCR**	100% (46/46)	100% (42/42)	100% (44/44)	100% (38/38)	
**(95% CI)**	92.29–100	91.62–100	91.97–100	90.82–100	

## Discussion

In this study, the Cygnus device was developed to detect DENV infection in plasma through viral NS1 antigen detection as well as DENV serotype identification simultaneously, without the use of complex laboratory equipment and with short time-to-result. The performance of the Cygnus device was evaluated with dengue clinical samples in comparison to an in-house serotyping-NS1 microplate ELISA which provides similar features for DENV NS1 detection and serotyping. Compared to a standard RT-PCR method, both assays showed similar sensitivity for the detection of NS1 regardless of serotype, 83% vs 82% for NS1-microplate ELISA and NS1-Cygnus respectively. Sensitivity to DENV1-4 serotypes compared to RT-PCR detected by NS1-Cygnus ranged from 76 to 80% with DENV3 being the most sensitive. While that of microplate NS1-ELISA showed a range of sensitivity from 76 to 92%, with DENV1 the most sensitive. (Table 6). Overall, specificity of NS1-microplate ELISA was higher than the NS1-Cygnus device, 100% vs 86%.

The anti-NS1 mAbs specific to each serotype as well as the pan-serotype mAb are critical reagents for the microcapillary based assay development and were selected based on high reactivity to direct NS1 coated ELISA and low K_D_ from surface plasmon resonance (S1 Table). The low specificity of the Cygnus test was a result of false positive samples assigned to DENV1, which was not found in NS1-microplate ELISA, although the same clones of serotype-specific mAbs were used. This might be explained by the different configuration of antibody pair which were designed for capture and detection between two assays. In the microplate ELISA, a greater flexibility in antibody pairings was possible, at the cost of needing separate plates and sample addition for each serotype assay. The pan-serotype antibody (clone 2E11) could be used as either the capture or detection antibody, but use as the capture antibody gave better performance, presumably because it could bind more effectively to epitopes shared between NS1 of all serotypes [22]. In contrast, the Cygnus platform, serotype-specific mAbs must be used as capture antibodies for multiplexing within each microcapillary test strip, while a pan-serotype mAb (clone 1F11) was needed for detection of all serotypes. This may lead to non-specific binding explaining those false positive cases. Furthermore, after assay optimization to produce the most sensitive assay, a higher concentration of DENV1 antibody (60 μg/ml) than that of other serotypes (40 μg/ml) was used to coat in microcapillary strips which may also increase the potential for background signal resulting in false positives. Noted in the NS1-microplate ELISA, this clone (84B) alone also gave poor sensitivity to DENV1 (31/50; 62%), but the sensitivity was enhanced to 92% by the addition of a further microplate assay using a DENV1/3 subcomplex antibody pair (clone 5F3) (Table 6 and S1 Data). From these lines of evidence, we suggest that the concept of the Cygnus to detect DENV NS1 and serotyping is promising, although DENV1 specific assay requires improvement.

Presently there are many commercial test kits to diagnose DENV NS1 by either microplate ELISA or rapid immunochromatographic LFT, for example, SD Bioline Dengue Duo (Alere, USA), Platelia NS1 Ag (Bio-Rad, France), or Panbio Dengue Early ELISA (Alere). Their sensitivity and specificity can vary widely (70–99%) [42,43]. However, some concerns remain about serological cross-reactivity between arboviruses such as newer Flavivirus including Zika that emerge in dengue endemic areas. One study used both SD Bioline Dengue Duo and Platelia Dengue NS1 Ag kit in 65 Zika-infected patients, but none of them showed positive results [44], suggesting high specificity of dengue NS1 assay. Nevertheless, none of the commercially available dengue NS1 test kits can differentiate DENV serotypes. In addition to conventional virus isolation and RT-PCR, in-house NS1 microplate ELISA can be used to differentiate serotypes [12,13,14,22,45]. Only one prototype of NS1 dipstick rapid test was recently developed for DENV serotyping with 76, 89, 100 and 100% sensitivity for DENV1-4 respectively and 89, 98, 100 and 96% specificity for DENV1-4, respectively [15].

Several studies have suggested that sensitivity of the NS1 assays in primary infection is greater than secondary infection cases in which more false negative samples were found [46,47,48,49]. Therefore, in DENV endemic areas where secondary infections are frequent, NS1 assay performance tends to be low. This is potentially due to the presence of NS1 immune complex (IC) which is generated by circulating NS1 and existing anti-NS1 antibodies [50,51,52] which reduces the amount of free NS1 protein. Disassociation of NS1 IC by heat or acid/base methods explored in previous studies [53,54,55] were found to enhance assay sensitivity. However, our NS1-Cygnus device and NS1-microplate ELISA gave above 82% sensitivity despite patient plasma used in this study being mostly from secondary infections (98%). This may suggest high binding ability of the selected anti-NS1 antibody pairs which were used in both assays, or high analytical sensitivity for both immunoassay platforms (microplate and microcapillary).

Regarding the development process of the Cygnus device, distinct from the flat plastic capillary film ELISA in previous studies [27,29,31], the Cygnus devices represent an improved and more accessible format for microfluidic immunoassays by coating with hydrophilic polymer (PVOH) allowing fluid flow to be driven entirely by gravity without requiring mechanical operation via the multi-syringe aspirator [31]. As found previously, the excellent optical transparency of fluorinated ethylene propylene (FEP) which has a refractive index equal to water, made it suitable for simple fluorescent substrate detection and straightforward recording of results by smartphone cameras [29]. No significant difference in analytical performance was seen between the different cameras used, confirming previous systematic comparison of cameras for microfluidic immunoassay detection [39]. The imaging processing for quantitation of NS1 levels, is currently performed manually. For greater uptake of the Cygnus test, this analysis should be automated, analogous to a lateral flow reader. Here, we demonstrated that this proof-of-concept test can be imaged using simple and low-cost optics with a smartphone camera, enabling the possibility of smartphone embedded app for automatic digital image analysis alongside scoring the test and reporting results electronically.

In summary, we demonstrate that our Cygnus prototype device may represent a promising tool for rapid serotype-specific detection of NS1 for diagnosis and surveillance of dengue infection in clinical samples. Due to its inexpensive mass-manufacture, portability, speed, and simplicity to operate, alongside sensitivity, specificity and accuracy that closely matches microplate ELISA for DENV2-4, we believe these devices could be used for surveillance and routine diagnosis worldwide especially in remote areas with limited access to laboratory infrastructure (e.g., microplate ELISA readers). This device could be helpful as an ancillary test alongside RT-PCR in hospital diagnostic labs or in the field. Moreover, it provides DENV serotyping information and rapid results that are valuable for real-time dengue epidemiology.

## Supporting information

S1 Fig Clinical specimens used for validation. (A) Schematic diagram of selected panels of clinical specimens. (B). Characteristics of dengue clinical samples.
(TIF)

S2 Fig Stepwise demonstration of the gravity driven MCF immunoassay. (A) Assay components. (B) Liquid reagents can be added by dropper bottles. (C) Multiple Cygnus cassettes can be moved as a block and (D) the microcapillary film interfaced with the custom stripwell. (E) Once the wells are empty of reagent the cassettes can be removed and (F-G) the stripwell replaced with a fresh one and (H) the next reagent can be added.
(TIF)

S1 Table Surface plasmon resonance results of monoclonal antibodies used in this study. (DOCX)

S2 Table DENV NS1 serotyping results by in-house microplate ELISA [22] from 255 samples are compared to RT-PCR. (DOCX)

S1 Data NS1 and serology results for 205 patient measured by Cygnus, microplate ELISA, RT-PCR and SD Bioline lateral flow. (XLSX)

S1 Text Detailed methodology for preparation and operation of Cygnus device. (PDF)

S1 STARD STARD checklist. (PDF)
