# Supplementary figures and images for: Smartphone multiplex microcapillary diagnostics using Cygnus: Development and evaluation of rapid serotype-specific NS1 detection with dengue patient samples
Source: PLoS Negl Trop Dis. 2022 Apr 7;16(4):e0010266. doi: 10.1371/journal.pntd.0010266 (PMC8989202; doi:10.1371/journal.pntd.0010266)

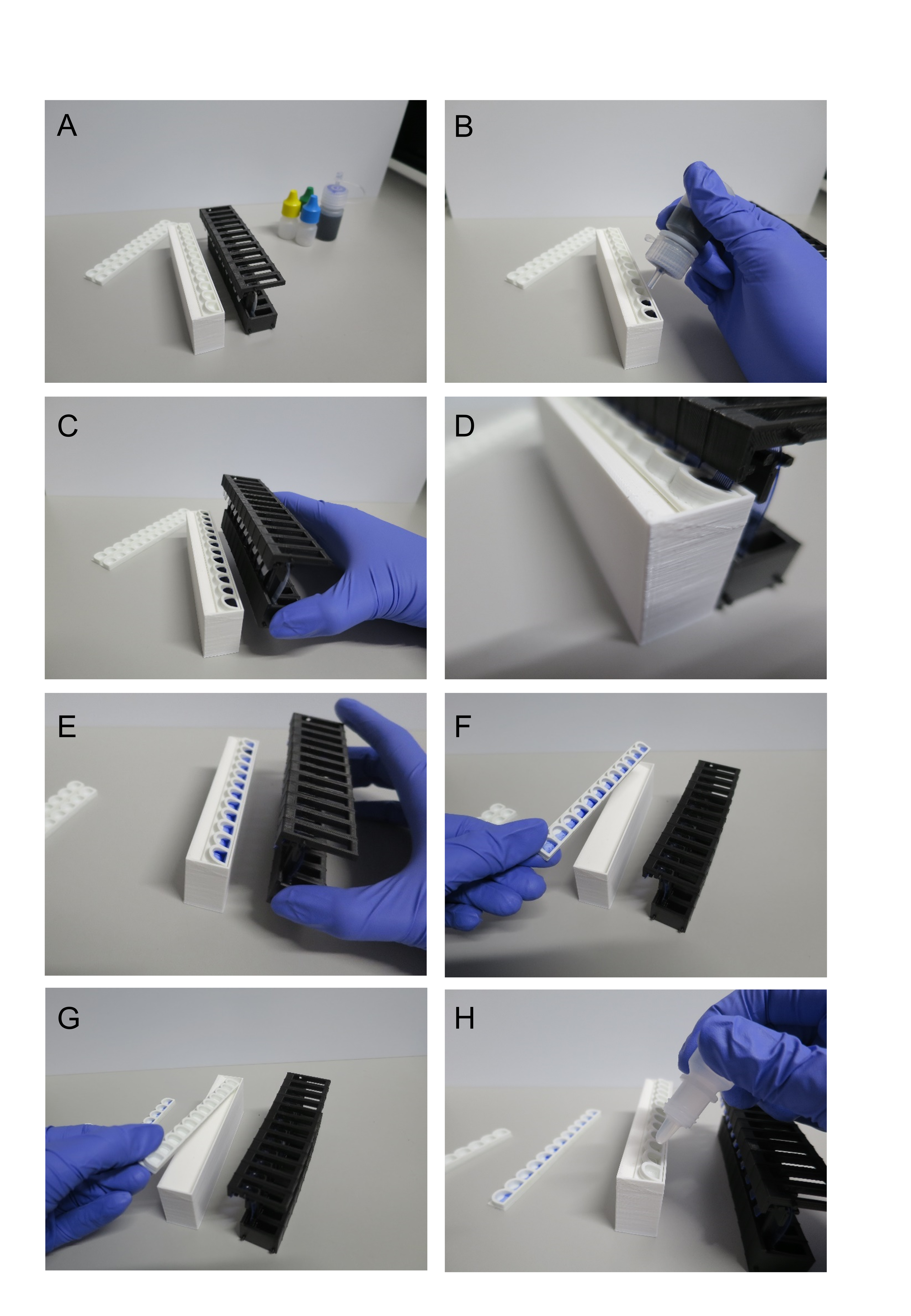

Supplement: S2 Fig — (A) Assay components. (B) Liquid reagents can be added by dropper bottles. (C) Multiple Cygnus cassettes can be moved as a block and (D) the microcapillary film interfaced with the custom stripwell. (E) Once the wells are empty of reagent the cassettes can be removed and (F-G) the stripwell replaced with a fresh one and (H) the next reagent can be added. (TIF) [file pntd.0010266.s002.tif]
