# Supplementary material for: Smartphone multiplex microcapillary diagnostics using Cygnus: Development and evaluation of rapid serotype-specific NS1 detection with dengue patient samples
Source: PLoS Negl Trop Dis. 2022 Apr 7;16(4):e0010266. doi: 10.1371/journal.pntd.0010266 (PMC8989202; doi:10.1371/journal.pntd.0010266)
Supplement: S1 Table — (DOCX) [file pntd.0010266.s003.docx]

**S1 Table.** **Surface plasmon resonance results of monoclonal antibodies use in this study.**

| **Ab** | **DENV1 NS1** | | | **DENV2 NS1** | | | **DENV3 NS1** | | | **DENV4 NS1** | | |
| --- | --- | --- | --- | --- | --- | --- | --- | --- | --- | --- | --- | --- |
|  | **K_ON_**  **(10^4^M^-1^s^-1^)** | **K_OFF_**  **(10^-4^s-^1^)** | **K_D_**  **(nM)** | **K_ON_**  **(10^4^M^-1^s^-1^)** | **K_OFF_**  **(10^-4^s^-1^)** | **K_D_**  **(nM)** | **K_ON_**  **(10^4^M^-1^s^-1^)** | **K_OFF_**  **(10^-4^s^-1^)** | **K_D_**  **(nM)** | **K_ON_**  **(10^4^M^-1^s^-1^)** | **K_OFF_**  **(10^-4^s^-1^)** | **K_D_**  **(nM)** |
| **84B** | 2.49±1.24 | 12.3±7.00 | 54.8±24.50 | - | - | - | - | - | - | - | - | - |
| **1B10** | - | - | - | 26.10±6.43 | 8.14±1.01 | 3.18±0.37 | - | - | - | - | - | - |
| **46A** | - | - | - | - | - | - | 21.30±16.26 | 20.50±10.24 | 12.30±7.00 | - | - | - |
| **4A** | - | - | - | - | - | - | - | - | - | 4.00±4.99 | 6.87±2.16 | 31.8±20.00 |
| **1F11** | 3.32±1.65 | 0.41±0.45 | 1.18±0.72 | 13.20±15.24 | 2.08±0.48 | 3.65±3.40 | 10±4.14 | 1.80±0.47 | 1.93±0.54 | 5.49±1.85 | 0.18±0.09 | 0.39±0.30 |
