# Supplementary material for: Smartphone multiplex microcapillary diagnostics using Cygnus: Development and evaluation of rapid serotype-specific NS1 detection with dengue patient samples
Source: PLoS Negl Trop Dis. 2022 Apr 7;16(4):e0010266. doi: 10.1371/journal.pntd.0010266 (PMC8989202; doi:10.1371/journal.pntd.0010266)
Supplement: S2 Table — (DOCX) [file pntd.0010266.s004.docx]

**S2 Table.** **DENV NS1 serotyping results by in-house ELISA [1] from 255 samples are compared to RT-PCR.**

| **stNS1-ELISA** | **RT-PCR**  **Positive** | | | | | **RT-PCR**  **Negative** | **% Serotype agreement to PCR** |
| --- | --- | --- | --- | --- | --- | --- | --- |
|  | **DENV1** | **DENV2** | **DENV3** | **DENV4** | **Total** |  |  |
| **DENV1** | 31 | 0 | 0 | 0 | 31 | 0 | 100% (31/31) |
| **DENV2** | 0 | 42 | 0 | 0 | 42 | 0 | 100% (42/42) |
| **DENV3** | 0 | 0 | 38 | 0 | 38 | 0 | 100% (38/38) |
| **DENV4** | 0 | 0 | 0 | 38 | 38 | 0 | 100% (38/38) |
| **DENV1 or 3** | 15 | 0 | 6 | 0 | 21 | 0 |  |
| **Negative** | 4 | 13 | 6 | 12 | 35 | 50 |  |
| **Total** | 50 | 55 | 50 | 50 | 205 | 50 |  |
| **Sensitivity to serotype (5 Abs)** | 92%  (46/50) | 76.4%  (42/55) | 88%  (44/50) | 76%  (38/50) |  |  |  |
| **Overall sensitivity** | 82.9% (170/205) | | | | | |  |
| **Overall specificity** | 100% (50/50) | | | | | |  |
| **%Dengue diagnosis accuracy** | 86.3% (220/255) | | | | | |  |

Reference

1. Prommool T, Sethanant P, Phaenthaisong N, Tangthawornchaikul N, Songjaeng A, Avirutnan P, et al. High performance dengue virus antigen-based serotyping-NS1-ELISA (plus): A simple alternative approach to identify dengue virus serotypes in acute dengue specimens. PLoS Negl Trop Dis. 2021;15(2):e0009065. Epub 2021/02/27. doi: 10.1371/journal.pntd.0009065. PubMed PMID: 33635874.
