# Supplementary material for: Smartphone multiplex microcapillary diagnostics using Cygnus: Development and evaluation of rapid serotype-specific NS1 detection with dengue patient samples
Source: PLoS Negl Trop Dis. 2022 Apr 7;16(4):e0010266. doi: 10.1371/journal.pntd.0010266 (PMC8989202; doi:10.1371/journal.pntd.0010266)
Supplement: S1 Text — (PDF) [file pntd.0010266.s006.pdf]

Supporting Information

Preparation of MCF multiplex test strips

MCF was produced by Lamina Dielectrics Ltd (Billingshurst, West Sussex, UK). It was made from FEP (Teflon, Dupont, USA) and controlled for size and shape through melt extrusion with air injection to incorporate microcapillaries. MCF was supplied in 100 m reels of flat plastic ribbon containing 10 parallel microcapillaries with an average internal diameter of 206±12.2 µm (Fig 1A). Capture antibodies were diluted in PBS and injected into individual capillaries using a 30G needle (Sigma Aldrich, UK) and incubated at room temperature for 1 hour to bind the internal surface by passive adsorption. The antibody solution was removed using a syringe to aspirate air through the capillaries, and replaced with 0.1% (w/v) polyvinyl alcohol (MW 146,000–186,000, > 99% hydrolyzed, Sigma Aldrich, Dorset, UK) in ultra-pure water at room temperature for 2 h (25) to create a hydrophilic internal surface. Coated MCF reels were then blocked with 4% (w/v) BSA (GE Healthcare Life Sciences, Pasching, Austria) overnight at 4 °C. To make the MCF test strip, coated reels were cut into pieces 47±1 mm long and placed into the Cygnus MCF holder cartridges (Fig 1B). The Cygnus holders, strip wells and strip well holder (Figs 1C-E) were 3D printed from PLA (Ooznest, UK) on a Prusa i3 MK3 3D printer (Prusa Research, Czech Republic), then washed before each use with detergent, 70% ethanol and thorough rinsing in water and air dried; holders were reused multiple times with no loss in performance.

Mouse monoclonal anti-DENV NS1 antibody clones which are serotype-specific were chosen from sets previously described and recently modified to be suitable for serotyping-NS1 ELISA (9, 17) while the pan-serotype DENV1-4 antibody clone was changed in this study. All clones are listed in Table 1. After monoclonal antibody (mAb) purification, the quality, reactivity, and quantity were examined by gel electrophoresis, western blot and NanoDrop spectrophotometry (Thermo Scientific, IL, USA), respectively. The pan-serotype DENV NS1

mAb was biotin labeled (EZ-Link NHS-Biotin; Thermo Scientific) and used as a common detection reagent in all assays. Serotype-specific DENV NS1 mAbs were coated onto the inner surface of MCF at 60 μg/ml for DENV1 and 40 μg/ml for DENV2-4 in duplicate capillaries at room temperature for 1 h. The last two capillaries were used as negative (PBS) and positive (recombinant DENV2 NS1 at 1μg/ml) controls, respectively (Fig 1G).

Enzyme amplified fluorescent ELISA in MCF platform using Cygnus devices

To perform the immunoassays, 60 μl of neat plasma was added into each sample well and a stack of MCF devices were interfaced with the well strip, allowing the samples to flow into each strip and then allowed to incubate for 10 min. The sequence of reagents was similar to conventional microplate ELISA but with significantly shorter incubation steps and is outlined in Fig 1F. Washing buffer (0.05% PBST+0.3 mg/ml of bromophenol blue) in a fresh strip of wells was run through the MCF test strips for 1 min. Incorporation of inert blue dye in the wash buffer allowed visual checking of effective washing and reagent addition. Biotinylated pan-serotype DENV NS1 antibody at 10 μg/ml and a 1:10 dilution of rat serum (to block non-specific background) was added and incubated in the MCF for 5 min followed washing and addition of a dilution 1:1000 of Streptavidin-AP (Sigma Aldrich, Dorset, UK) for 5 min. Lastly, a series of three replicate washes was followed by addition of AttoPhos® (Promega, WI, USA) substrate and incubated to allow enzyme amplification of NS1 detected, with images taken after 5, 10 and 15 minutes. The green fluorescence was examined under a blue LED light excitation, imaged through an amber emission filter using blue light transilluminator (IORodeo, USA). The image was recorded by digital camera (Canon S120) and smartphone camera (iPhone 6 and Sony Xperia) within 15 min of substrate addition. Fluorescein at 0, 1, and 5 mM were freshly prepared and used for fluorescent signal normalization by imaging samples alongside reference strips of MCF containing these three

dye concentrations, permitting direct comparison of signal intensities between different images and experiments even if camera image properties fluctuated.
